# Sex Pheromone of the Click Beetle *Agriotes pilosellus* (Schönherr, 1718)

**DOI:** 10.1007/s10886-021-01346-y

**Published:** 2022-01-24

**Authors:** Till Tolasch, Maximilian von Fragstein, Johannes L. M. Steidle

**Affiliations:** Institut für Biologie, Universität Hohenheim, Chemische Ökologie 190t, Garbenstraße 30, 70593 Stuttgart, Germany

**Keywords:** *Agriotes pilosellus*, Coleoptera, Elateridae, Sex pheromone, Geranyl butanoate, (*E*)-8-Hydroxygeranyl dibutanoate, Monitoring

## Abstract

*Agriotes pilosellus* is a fairly common click beetle species distributed in open deciduous and mixed forests throughout a large area in Europe. To identify its sex pheromone, gland extracts of female beetles were analyzed using gas chromatography-mass spectrometry (GC-MS). The only volatile compounds present in the extracts were geranyl butanoate and (*E*)-8-hydroxygeranyl dibutanoate in a 1:3 ratio, identified by comparison with synthetic samples. Field experiments revealed a clear attraction of *A. pilosellus* - males towards traps baited with geranyl butanoate, which could be synergistically enhanced by the factor of almost ten by addition of (*E*)-8-hydroxygeranyl dibutanoate. The latter compound alone did not show any attractive effect. Both compounds correspond well to the structures known from other *Agriotes* species and may serve as an effective monitoring tool for entomofaunistic research.

## Introduction

With a body length of 13-17 mm, the click beetle *Agriotes pilosellus* is the largest of the 16 central European *Agriotes* species. Its brownish black body is covered with a dense grey pubescence. With its unusual slender habitus and the pointed and diverging hind angles of the pronotum this click beetle strikingly differs from its congeners, resembling the closely related *Ectinus aterrimus* (L.) instead (Lohse 1979).

*Agriotes pilosellus* is mainly a European species, distributed over central and south Europe from Spain to Turkey (Laibner 2000; Cate 2007). Northwards, records become more and more scattered. It is considered scarce in northern Germany (Horion 1953), vulnerable in Denmark (Wind and Pihl 2010), and extinct in Sweden (Lundberg and Gustafsson 1995). The species is absent in the other Scandinavian countries, the Baltic states, and Great Britain.

In central Europe, *A. pilosellus* is fairly common in open deciduous and mixed forests, especially on their edges and on glades, from lowlands to the submontane zone. The larvae of *A. pilosellus* develop in forest soil for several years, with pupation taking place between July and August (Beling 1886; Korschefsky 1941). Adults remain in their pupal cells during their final hibernation to then emerge in late April. They can be found in the vegetation until July when the flight season usually ends.

*Agriotes pilosellus* has been said to cause damages on carrots, grain, and potatoes (Rudolph 1974), however, it is very likely that this information is based on misidentification, because open agricultural habitats are largely unsuitable for the species.

Here, the identification and synthesis of the sex pheromone of *Agriotes pilosellus* are reported. Sex pheromones of click beetles are generally female-produced and attract conspecific males, often over long distances and in large numbers. So far, fewer than 40 species have been investigated for their pheromones, which corresponds to less than 0.5 % of the known click beetle species. Most of them are other members of the genus *Agriotes*, which includes some economically important pest species, and which mainly use geranyl- and/or (*E*,*E*)-farnesyl esters of acids with an even number of carbons (two to eight) as sex pheromones (for an overview, see Tóth 2013). The present study aimed to expand the knowledge of the pheromones of central European *Agriotes* species and to provide an effective monitoring tool for entomofaunistic purposes.

## Methods and Materials

### Insects and Extracts

Adult beetles of *A. pilosellus* were collected from beech twigs using a beating tray. Beetles were sexed by the longer antennae and the more slender habitus of the males. A total of six females were taken from two different field sites in Baden-Württemberg/Southwest Germany: A mixed forest near Göppingen (N 48.751°, E 9.586°, WGS 84) in June 2004 and a beech forest near Owen/Teck (N 48.582°, E 9.468°) in May 2005.

Beetles were euthanized by deep freezing them in the lab (-22°C) for at least 30 minutes, then individually allowed to thaw for another 10 minutes. Subsequently, the ovipositor was carefully extricated from the abdomen with forceps, and the now visible paired pheromone glands, which were found to be similar to those in other members of the subfamily Elaterinae (Merivee and Erm 1993; Tolasch et al. 2007, 2010, 2013) were excised. One extract of three females was obtained by transferring their glands into 300 μl dichloromethane, the remaining three females were treated separately by extracting their glands with 100 μl dichloromethane each.

### Chemical Analyses

Analyses of the natural extracts were carried out by coupled gas chromatography–mass spectrometry (GC/MS) employing a 6890 GC gas chromatograph linked to a 5973N MSD (both Agilent Technologies, Santa Clara, CA, USA) in electron impact mode at 70 eV. By use of helium as the carrier gas (1.2 ml/min), compounds were separated on an HP-5MS column (30 m x 0.25 mm ID, 0.25-mm film thickness; Agilent) that was operated at 60°C for 3 min, increased to 300°C at a rate of 3°C /min, and finally held at this temperature for 10 min. Compounds were identified by comparison of mass spectra and retention indices with those of authentic standards. Linear retention indices (RI) for compounds were determined under the same conditions by comparing retention times with those of a homologous series of *n-*alkanes (C8–C30). Nuclear magnetic resonance (NMR) spectra of synthetic compounds were recorded with a Bruker DRX500 instrument (Bruker, Rheinstetten, Germany).

### Synthesis

Chemicals (geraniol, butyryl chloride, selenium dioxide, and *tert*-butyl hydroperoxide) were purchased from Sigma-Aldrich (Schnelldorf, Germany), solvents from Carl Roth (Karlsruhe, Germany) or Merck (Darmstadt, Germany) and were of the highest purity available. Purification of synthetic products was carried out by flash chromatography on silica gel (silica 32-63, 60 Å, ICN-Biomedicals, Eschwege, Germany) at 1.3 bar using mixtures of ethyl acetate and hexane.

Geranyl butanoate (**1**) was prepared from geraniol and butyryl chloride according to standard methods (Tóth et al. 2003). Compound **1** was oxidized to (*E*)-8-hydroxygeranyl butanoate using selenium dioxide and *tert*-butyl hydroperoxide, following reported procedures (Arm et al. 1986). Esterification of this product with butyryl chloride gave (*E*)-8-hydroxygeranyl dibutanoate ((2*E*,6*E*)-2,6-dimethyl-2,6-octadien-1,8-diyl dibutanoate, **2**) in a total yield of 63%.

1H-NMR (CDCl_3_, 500 MHz): δ (ppm) = 0.95 (2t, *J* = 7.4 Hz, 6H, C-4 and C-4”), 1.60-1.71 (m, 4H, 3-H and 3”-H), 1.65 (s, 3H, 7’-CH_3_), 1.71 (s, 3H, 3’-CH_3_), 2.05-2.10 (m, 2H, 4’-H), 2.13-2.20 (m, 2H, 5’-H), 2.28 (2t, *J* = 7.4 Hz, 4H, 2-H and 2”-H), 4.45 (s, 2H, 8’-H), 4.59 (d, *J* = 6.9 Hz, 2H, 1’-H), 5.35 (t, *J* = 7.1 Hz, 1H, 2’-H), 5.43 (t, *J* = 6.9 Hz, 1H, 6’-H).

^13^C-NMR (CDCl_3_, 101 MHz): δ (ppm)= 14.07 (2q, C-4 and C-4”), 14.38 (q, 7’-CH3), 16.86 (q, 3’-CH_3_), 18.89 (2t, C-3 and C-3”), 26.38 (t, C-5’), 36.67 (2t, C-2 and C-2”), 39.28 (t, C-4’), 61.40 (t, C-1’), 70.24 (t, C-8’), 119.27 (d, C-2’), 129.18 (d, C-6’), 131.00 (s, C-7’), 141.90 (s, C-3’), 173.59 (2s, C-1 und C-1”). Carbon numbers of the acid moieties in position 1 are indicated by one apostrophe, in position 8 by two apostrophes. MS (70 eV): See Fig. 2.

### Bait Dispensers and Traps

Dispensers were prepared from 0.2 ml PCR tubes (ThermoTube™, Peqlab, Germany) as described earlier (Tolasch et al. 2007). Synthetic test substances were applied as *n*-hexane solutions (100 mg/ml) into the tubes, which were pierced twice directly before use with an insect pin (diameter 0.5 mm) at the front side 2 mm below the lid. Dispensers were baited with one of the three lures: **A**: 4 mg geranyl butanoate (**1**), **B**: 12 mg (*E*)-8-hydroxygeranyl dibutanoate (**2**), and **C**: 4 mg **1** + 12 mg **2**, resembling the natural ratio of 1:3 found in the glands. Under laboratory conditions at room temperature, such odor dispensers with similar substances had a stable mean release rate of approximately 8–10 μg per day, regardless of the amount in the tubes (Tolasch et al. 2010).

Funnel traps were the same as described earlier (Tolasch et al. 2007). Each collecting bottle was filled with brine (250 ml) to euthanize and preserve the beetles and minimize the possible attraction of further individuals to those already captured.

## Field Experiments

Two separate field experiments were run in 2006 and 2012. The first experiment was designed to test the general attractive effect of **1** and **2** in a blend lure resembling the natural ratio found in the glands. It was carried out from 4-13 May 2006 in the same mixed forest near Göppingen, where a part of the females had been collected (see above). Predominant trees were spruce (*Picea abies* (L.) Karst) and beech (*Fagus sylvatica* L.) with single oaks (*Quercus robur* L.) and birches (*Betula pendula* Roth) interspersed. Ten sets of paired traps, one trap in each pair baited with lure **C** and the other left unbaited, were suspended ca. 1.5–2.0 m above the ground from branches at clearings and along forest roads. The distance between traps within one set was about 5 m, while the distances between the sets were at least 50 m. Traps were checked once after four days, captured beetles were removed and the brine was replaced, and removed five days later.

The second experiment in 2012, designed to examine the activity of the single compounds vs. the mixture, was performed from 2-31 May 2012 in a beech forest near Owen/Teck, the second locality where samples had been taken (see above). Forty traps were grouped into ten sets, each set comprised of three baited traps with lure **A**, **B**, and **C**, respectively, and an empty control. Traps were suspended as described above, but the distances between the traps were larger (> 10 m within one set, 100-200 m between the sets). Traps were checked weekly, captured beetles were removed and the brine was replaced. Trap positions were not altered during the experiments.

## Statistics

The numbers of male beetles caught per time and trap were analyzed using the Software R 3.3.2 (R Core Team 2016). Data were checked for normal distribution and homogeneity of variances using Shapiro-Wilk normality test and Levene's Test for Homogeneity of Variance, respectively. Because data were not normally distributed and due to inhomogeneity of variances, beetle numbers in traps from the first experiment with lure **C** and the unbaited control were compared using Wilcoxon rank sum test with continuity correction. For the second experiment, we calculated a generalized mixed model (family “negative binomial”), with trap set number and 7-day period as a random variable (Bates et al. 2015) followed by post-hoc Tukey’s tests (Hothorn et al. 2008).

## Results

### Chemical Analyses

**Fig. 1:**
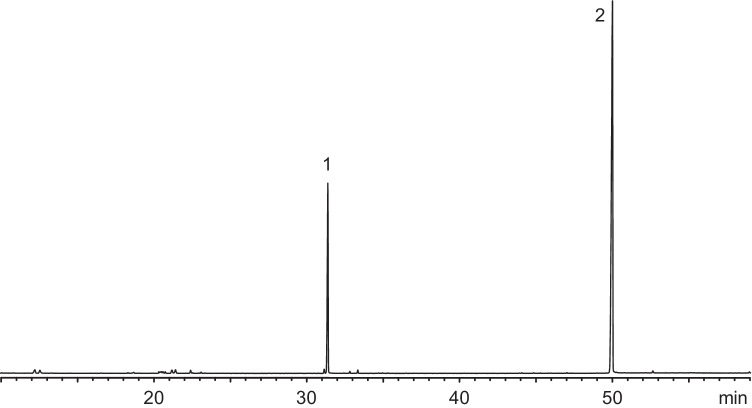
Representative gas chromatogram (total ion current) of a pheromone gland extract of a single *Agriotes pilosellus* female. Compound numbers correspond to the text and Fig. 3 (30 m HP-5MS capillary column, 3 min at 60°C, then 3°C/min to 300°C)

GC/MS analysis revealed two compounds present in all gland extracts at a ratio of ﻿almost exactly 1:3, with hardly any quantitative variation between the samples of different females (Fig. 1). The minor compound, eluting at 31.4 min (RI 1562) under the conditions described, was identified as geranyl butanoate (**1**) by comparison with a synthetic sample (median: 25.48 % TIC area, min: 23.27 %, max: 26.94 %, N=4).

**Fig. 2:**
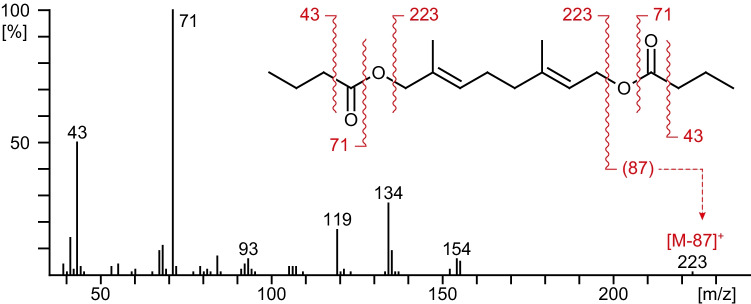
70 eV–mass spectrum of the second *Agriotes pilosellus* gland extract compound eluting after 49.9 min (RI 2112), subsequently identified as (*E*)-8-hydroxygeranyl dibutanoate (2)

**Fig. 3:**
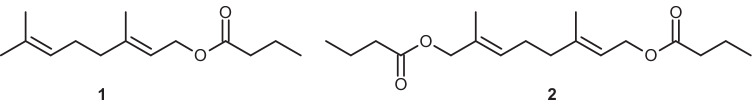
Compounds identified from pheromone gland extracts of female *Agriotes pilosellus*: Geranyl butanoate (1) and (*E*)-8-hydroxygeranyl dibutanoate (2)

The major compound at 49.9 min (RI 2112) exhibited a terpenoid mass spectrum (*m/z* 134, 152, and 154, Fig. 2), strongly resembling the spectrum of (*E*)-8-hydroxyneryl dihexanoate known from *Agriotes acuminatus* (Tolasch et al. 2010). The most obvious difference was the base peak *m/z* 71, instead of *m/z* 99, indicating the corresponding dibutanoate. The compound was subsequently identified as (*E*)-8-hydroxygeranyl dibutanoate (**2**) (median: 74.52 %, min: 73.06 %, max: 76.73 %, N=4). The structures of both gland compounds are shown in Fig. 3.

### Field Trapping Experiments

**Fig. 4:**
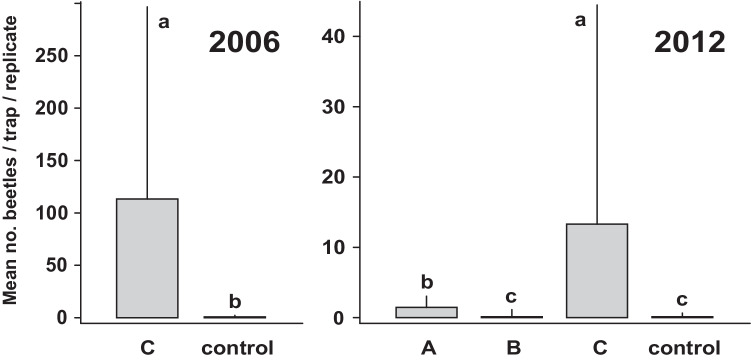
Mean (+ SE) number of male *Agriotes pilosellus* caught in traps baited with **A**: 4 mg geranyl butanoate (**1**), **B**: 12 mg (*E*)-8-hydroxygeranyl dibutanoate (**2**), or **C**: **A** + **B** in 2006 (N=20) and 2012 (N=34). Columns with different lowercase letters are significantly different at P<0.001 (Left: Wilcoxon rank sum test with continuity correction; right: Generalized mixed model (family “negative binomial”) followed by Tukey tests

In the first field experiment, carried out in 2006, a 1:3 mixture of **1** and **2** (bait **C**) proved to be highly attractive for swarming males of *Agriotes pilosellus*. A total of 2217 ♂♂ were caught in the traps within nine days, with only 12 ♂♂ found in the empty control traps, showing a highly significant difference (Wilcoxon rank sum test with continuity correction: W = 8.5, *p* = 1.388 x 10^-7^, Fig. 4, 2006, left).

The results of the second field test (2012), designed to investigate the activity of the single compounds **1** and **2** vs. the mixture, are shown in Fig. 4 (2012, right). This test revealed highly significant differences among the different types of baits (Chi^2^ = 112.02; df = 3: *p* = 2.2 x 10^-16^ ***). Geranyl butanoate (**1**, bait **A**), showed a moderate but clear attractiveness with a capture of 48 males in total (Tukey test control vs. **A**: *p* < 0.001 ***). Catches with the mixture (bait **C**) were almost 10 times higher (451 ♂♂), showing a significant difference and a strong synergistic effect of **2** towards geranyl butanoate (**1**) (Tukey test **C** vs. **A:** *p* < 0.001 ***). Compound **2** alone (Lure **B**) did not show an attractive effect, the total catches (9 ♂♂) were not significantly different from the numbers in the control traps (6 ♂♂) (Tukey test control vs. **B**: *p* < 0.986 n.s.). Because single traps were damaged or vanished during the experiment, the number of replicates had to be reduced from 40 to 34. No female *A. pilosellus* were caught in the experiments.

## Discussion

The results of our study clearly show that the sex pheromone of *Agriotes pilosellus* consists of geranyl butanoate (**1**) and (*E*)-8-hydroxygeranyl dibutanoate (**2**) in a ratio of 1:3. While **1** is necessary to generate attraction at all, **2** acts synergistically and enhances the activity considerably. As a single compound, **2** did not show any attractive effect. Both compounds identified correspond well to the structures known from other *Agriotes* species, which are generally made up of one or two esters of acyclic isoprenoid alcohols with short fatty acids (two to eight carbons).

Geranyl butanoate (**1**) has already been described as a pheromone component in several *Agriotes* spp. (reviewed in Tóth 2013), among them economically important species like *A. sputator* (Yatsynin et al. 1986; Kudryavtsev et al. 1993; Siirde et al. 1993) and *A. brevis* (Tóth et al. 2002). The second compound in *A. pilosellus*, (*E*)-8-hydroxygeranyl dibutanoate (**2**), is less well known. As a trace compound and without any recognizable function, it has so far only been found in the Australian shield bug *Oechalia schellenbergii* (Guérin-Méneville) (Aldrich et al. 1996), and in the click beetle *Agriotes brevis* (Tolasch and Tóth, unpublished). The alleged occurrence in *Agriotes modestus* Kiesenwetter (= *A. ponticus* Stepanov), quoted by Tóth (2013), is apparently erroneous, as in fact the (*Z*)-2-configurated (*E*)-8-hydroxyneryl dibutanoate is mentioned in the original work (Yatsynin et al. 1996).

Pheromone systems similar to the one reported for *A. pilosellus*, consisting of an acyclic monoterpene ester as an essential compound and a diester with the same monoterpene skeleton as an enhancing synergist, have already been found in two other *Agriotes* species: The south-east European *A. tauricus* uses geranyl isovalerate as a necessary compound, enhanced by (*E*)-8-hydroxygeranyl diisovalerate (Yatsynin and Rubanova 1983; Kudryavtsev et al. 1993), while the attractive effect of neryl butanoate in the central European *A. acuminatus* can be multiplied by the addition of (*E*)-8-hydroxyneryl dihexanoate (Tolasch et al. 2010). Consequently, the pheromone composition of *A. pilosellus* described in the present study seems to integrate well into the general scheme of biologically active terpene esters of the genus *Agriotes*.

As mentioned in the introduction, *A. pilosellus* is not a pest species and does not cause economical losses in forestry or agriculture. On the other hand, sex pheromones have been used successfully for recording rare click beetle species like *Betarmon bisbimaculatus* (König et al. 2016) or *Idolus picipennis* (Tolasch et al. 2013). The synthetic sex pheromone of *Agriotes pilosellus* may also serve as an effective monitoring tool for obtaining faunistic records where the species is rare, reaches the limit of its known distribution area or is considered extinct.

## Data Availability

All relevant data included in the article.
